# Potassium channels as a potential therapeutic target for trigeminal neuropathic and inflammatory pain

**DOI:** 10.1186/1744-8069-7-5

**Published:** 2011-01-10

**Authors:** Mamoru Takeda, Yoshiyuki Tsuboi, Junichi Kitagawa, Kazuharu Nakagawa, Koichi Iwata, Shigeji Matsumoto

**Affiliations:** 1 Department of Physiology, School of Life Dentistry at Tokyo, Nippon Dental University, 1-9-20, Fujimi-cho, Chiyoda-ku, Tokyo,102-8159, Japan; 2 Department of Physiology, School of Dentistry, Nihon University, 1-8-13, Kandasurugadai, Chiyoda-ku, Tokyo, 101-8310, Japan; 3 Division of Oral Physiology, Department of Oral Biological Science, Niigata University Graduate School of Medical and Dental Sciences, 2-5274, Gakkocho-dori, Niigata, 951-8514, Japan; 4 Department of Hygiene and Oral Health, Showa University School of Dentistry, 1-5-8, Hatanodai, Shinagawa-ku, Tokyo, 142-8555, Japan

## Abstract

Previous studies in several different trigeminal nerve injury/inflammation models indicated that the hyperexcitability of primary afferent neurons contributes to the pain pathway underlying mechanical allodynia. Although multiple types of voltage-gated ion channels are associated with neuronal hyperexcitability, voltage-gated K^+ ^channels (Kv) are one of the important physiological regulators of membrane potentials in excitable tissues, including nociceptive sensory neurons. Since the opening of K^+ ^channels leads to hyperpolarization of cell membrane and a consequent decrease in cell excitability, several Kv channels have been proposed as potential target candidates for pain therapy. In this review, we focus on common changes measured in the Kv channels of several different trigeminal neuropathic/inflammatory pain animal models, particularly the relationship between changes in Kv channels and the excitability of trigeminal ganglion (TRG) neurons. We also discuss the potential of Kv channel openers as therapeutic agents for trigeminal neuropathic/inflammatory pain, such as mechanical allodynia.

## Introduction

Pain caused by a lesion of the peripheral or central nervous system is commonly termed neuropathic pain, and this type of pain frequently persists, even following normal repair of the injured tissue [1-3]. In a clinically significant proportion of cases, the neuropathic pain becomes chronic, severely debilitating, and extremely difficult to treat. Although several different types of neuropathic pain animal models have been developed and extensively studied [4,5], no common therapeutic molecular target has been identified for neurons located in the nociceptive pathway.

Multiple types of voltage-gated ion channels are associated with neuronal excitability. Among these, voltage-gated K^+ ^(Kv) channels are important physiological regulators of membrane potentials, action potential shape, and firing adaptation in excitable tissues including nociceptive sensory neurons [6-8]. Dorsal root ganglion (DRG) and trigeminal ganglion (TRG) neurons expressed three distinct classes of K^+ ^currents in varying quantities: slow-inactivating sustained (K-current; I_K_), fast-inactivating transient (A-current; I_A_), and slow-inactivating transient (D-current; I_D_) currents based on their inactivation of kinetics and sensitivities to tetraethylammoniun (TEA), 4-aminopyridine (4-AP) and α-dendorotoxin (α-DTX), respectively [9-13]. Peripheral nerve injury and inflammation markedly reduces the densities of Kv channels, implicating them in the development of neuropathic/inflammatory pain [14-17]. Since the opening of K^+ ^channels leads to hyperpolarization of cell membrane and a resultant decrease in cell excitability, several types of Kv channels have been proposed as potential target candidates for such pain pathways [18]. Recent studies in various pain models identified the voltage-gated KCNQ/Kv7 channel (M-current) opener [19-21] and alterations in either calcium-activated (K_Ca_) or ATP-sensitive potassium (K_ATP_) channels as potential therapeutic targets for neuropathic/inflammatory pain [22,23].

We recently reported on the hyperexcitability of primary afferent neurons in several different trigeminal nerve injury and inflammation animal models, including chronic constriction nerve injury (CCI), axotomy, and inflammatory models [17,24-26]. In these models, we systemically investigated the mechanism of mechanical allodynia underlying changes in the TRG neuronal excitability due to several types of neuropathic/inflammatory conditions in rats, using behavioral analysis as well as extracellular single-unit and whole-cell patch-clamp recordings.

This review therefore focuses on changes in the Kv channels studied in different trigeminal neuropathic/inflammatory pain animal models, and particularly the relationships between Kv channels and TRG neuron excitability. We also discuss potential therapeutic targets identified thus far for the prevention of pathological pain, such as those targeting mechanical allodynia.

### Pathological pain models

Peripheral nerve injury/inflammation produces sensory abnormalities associated with chronic pain. Peripheral tissue inflammation can alter the property of somatic sensory pathways, resulting in behavioral hypersensitivity due to the increased responses to pain caused by noxious stimulation (hyperalgesia) as well as the normally non-noxious stimulation (allodynia). Several different types of animal nerve injury models have been developed to elucidate the neuronal mechanisms underlying these abnormal pain sensations (allodynia and hyperalgesia) [27-29]. Among these models, the CCI model shows inflammation and nerve injury, and can mimic neuropathic pain in human [27,30]. CCI of the sciatic nerve is indeed one of the most reliable models of neuropathic pain [31]. Following peripheral nerve injury, primary afferent neurons exhibit high frequency background activity or irregular burst discharges [27]. On the other hand, in the axotomy of primary afferent neuron model (*axotomy model*), the hyperexcitability of primary afferent neurons induced by peripheral nerve injury is thought to result from extensive changes in the ionic currents of DRG neurons [32,33]. Moreover, changes in the excitability of primary afferent neurons were also observed at the adjacent intact primary afferent neurons after nerve injury (*axotomy spared neuron models*) [34,35].

Any discussion of trigeminal neuropathic pain should consider the following points. First, the trigeminal nerve has some unique characteristics that may influence its response to injury, such as its embryological origin, and the proportion of myelinated and unmyelinated nerves, and sympathetic fibers [36-38]. In addition, the location of some branches of the nerve within bony canals protects them from exogenous stimuli, but makes them vulnerable to pressure (e.g., edema or displacement of fractured bone fragments) [36]. To elucidate the mechanism underlying trigeminal mechanical allodynia in different neuropathic pain/inflammatory models, we recently investigated four such animal models, as described in the following sections.

### Changes in potassium currents and trigeminal neuropathic and inflammatory pain

**Figure 1:**
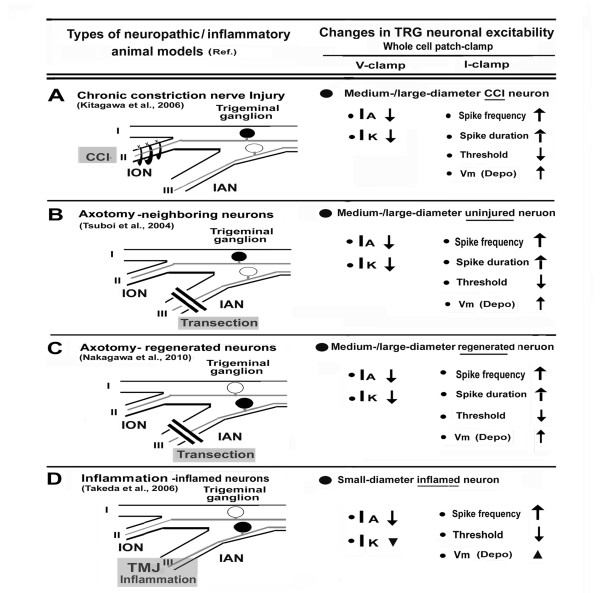
**Trigeminal neuropathic/inflammatory pain models and changes in TRG neuronal activities**. ***A***: *Chronic constriction nerve injury (CCI) model*. Three days after infraorbital nerve (ION) chronic constriction. Target trigeminal ganglion (TRG) neurons were labeled by fluorogold (FG). Whole-cell patch-clamp recordings under current-/voltage-clamp configurations were performed on medium/large-diameter CCI neurons. ***B***: *Axotomy-neighboring neuron model*. In this model, we found mechanical allodynia in the territory of ION at 2 days after inferior alveolar nerve (IAN) transection. Uninjured TRG neurons innervating the territory of ION were labeled by FG and patch-clamp recordings under current-/voltage-clamp configuration was performed on medium-/large-diameter neurons. ***C***: *Axotomy-regenerated neuron model*. In this model, we observed mechanical allodynia at 14 days after IAN transection. FG-injection at 14 days after IAN transection showed massive labeling of trigeminal ganglion containing FG-labeled small/large-diameter neurons and the patch-clamp recordings under current-/voltage-clamp configuration indicated axotomy-regenerated neurons. ***D***: *Inflammation model*. Inflammation was induced by injection of CFA into the rat temporomandibular joint (TMJ). We found mechanical allodynia in the territory of ION at 2 days after CFA injection. In FG-labeled small-diameter TRG neurons innervating TMJ, the whole-cell patch-clamp recordings under current-/voltage-clamp configurations were performed on inflammatory neurons. Blackened circles indicate the trigeminal ganglion neurons. I-III: trigeminal nerves, I_A_: transient K current, I_K_: sustained K currents, ↑: increase, ↓: decrease, ▼: tendency for decrease in I_K_, but not significant, ▲: tendency for depolarization of membrane potential (Vm), but not significant, Depo: Depolarization, V-clamp : Voltage-clamp, I-clamp: Current-clamp.

Figure 1 summarizes the changes in TRG neuronal excitability associated with the changes in Kv channels using whole-cell patch-clamp recording in our trigeminal neuropathic/inflammatory pain animal models [17,24-26]. In each animal model, mechanical allodynia was determined by withdrawal threshold for the mechanical stimulation of the whisker pad area.

#### CCI-model

Trigeminal nerve has a predominant sensory function compared with that of other peripheral nerves such as sciatic nerve because it is predominantly composed of sensory afferents, and only contains a small number of efferent fibers. It can be assumed that the infraorbital nerve (ION) does not contain any motor fibers, thus the trigeminal CCI model provides evidence to demonstrate the neuropathic pain mechanism following a purely sensory nerve injury [25]. In our study, mechanical allodynia appeared at three days after ION-CCI and lasted for 14 days. Whole-cell patch-clamp recordings from medium-/large-diameter CCI-TRG neurons revealed that both I_K _and I_A _in rats with ION-CCI were significantly smaller than in naïve rats (Figure 1A). Changes in the duration of the depolarization phase (DDP) and repolarization phase (DRP) contribute to the I_A _and I_K, _respectively [12], thus suppression of these current in TRG neurons following CCI correlated with a decrease in DDP and an increase in DRP, respectively. Also, the TRG neuronal action potentials (and consequently, the total action potential duration) in rats with ION-CCI were significantly prolonged than those in naïve rats. Under current-clamp experiments, TRG neurons following ION-CCI showed a significant decrease in threshold currents for generating spike and a depolarizing effect of resting membrane potentials. In addition, depolarizing pulses following ION-CCI significantly increased the discharge frequencies of the TRG neuronal action potentials. These changes in medium-/large-diameter CCI-TRG neurons could contribute to the development of mechanical allodynia.

#### Axotomy-spared neuron model

Uninjured TRG neurons show similar alterations to adjacent TRG neurons following injury [24,34]. In this same model, we found that mechanical allodynia in the territory of ION was observed at 3 days after inferior alveolar nerve (IAN) transection. As shown in Figure 1B, voltage-clamp recording indicated that both I_K _and I_A _in the medium-/large-diameter spared (uninjured) TRG neurons innervating second branches of the trigeminal nerve (ION) in rats with IAN were significantly smaller than those in naïve rats, as for the CCI model. Corresponding with these changes, we also measured the following changes under current-clamp conditions: *1) *frequencies of the spike discharge induced by depolarizing pulses and duration of uninjured TRG neurons in rats with IAN were significantly increased; and *2) *significant decrease in threshold current for generation of spike and a depolarizing effect of resting membrane potentials of spared TRG neurons after IAN transection. These changes of medium-/large-diameter spared TRG neurons might contribute to the development of mechanical allodynia through paracrine signaling (e.g., brain-derived neurotrophic factor) from adjacent injured TRG neurons [24].

#### Axotomy-regenerated neuron model

Injured nerves regenerate several weeks after nerve damage [39-41]. Some clinical reports have noted that areas innervated by the regenerated nerve show an altered sensitivity to a variety of stimuli compared to those innervated by the intact nerve activity [42-45]. The regenerated neurons also exhibit ectopic discharges [46,47]. Recently we investigated the neural mechanisms underlying abnormal pain following regeneration of the injured IAN [26]. In this model, we observed mechanical allodynia at 11-14 days after IAN transection. Injection of the retrograde tracer, fluorogold (FG), at 14 days after IAN transection showed extensive labeling of TRG neurons, suggesting that most of the TRG neurons present at that stage were regenerated. Whole-cell patch-clamp recording of FG-labeled small-/medium-diameter reinnervated TRG neurons showed a significant decrease in both I_A _and I_K _in the TRG neurons, a response that was associated with an increase in the spike generation, resulting in the hyperexcitability of reinnervated IAN-TRG neurons (Figure 1C). Taken together with previous results from intracellular single-unit recording [26], these changes may be due to the mechanical allodynia.

#### Inflammation-model

Complete Freund's adjuvant (CFA) models of inflammation in the orofacial region have been developed in rats to study the trigeminal nervous system [48,49]. Temporomandibular joint (TMJ) inflammation is associated with spreading pain and hyperalgesia [50] and TMJ disorder paitients complain of pain from innoxious vibrotactile stimulation [51]. We recently reported that TMJ inflammation modulates the excitability of Aβ-TRG neurons innervating the facial skin via a paracrine mechanism due to the release of substance P (SP) from the small-diameter TRG neurons. Such a release may be important in determining the trigeminal inflammatory allodynia associated with TMJ disorders [52,53]. Using this TMJ inflammatory model, we found that mechanical allodynia in the whisker skin region and I_A _densities in the small-diameter TRG neurons innervating TMJ were both significantly decreased in inflamed rats compared to naïve rats [17]. TMJ inflammation significantly reduced the threshold current and significantly increased action potential firing evoked by depolarizing current pulses. In this model there is a tendency for decreases in I_K _density in TMJ inflamed rats compared to naïve rats, although the change is not significant. Since application of 4-aminopyridine (A-type K channel blocker) to naïve rats TRG neurons mimicked the changes in the firing properties observed after CFA treatment [17], I_A _suppression of the TRG neurons innervating the TMJ could affect trigeminal inflammatory allodynia in TMJ disorders via paracrine mechanisms of SP in the trigeminal ganglia.

### Potassium channels as a potential target for trigeminal neuropathic/inflammatory pain

Lawson et al. [18] reported that several types of Kv channels have been proposed as potential target candidates for pain therapy. It was recently demonstrated that a selective KCNQ/Kv7 channel opener (retigabine), which mediates M-currents, selectively reduces the activity of axotomized Aδ/C-fibers, but not uninjured axons [19], and human C-fiber axons [21]. Xu et al. [20] also demonstrated that this KCNQ/Kv7 channel opener could attenuate allodynia due to TMJ inflammation in rats. Similarly, axotomy reduces K_Ca _channel activity in the small to medium sized DRG neurons, which would also increase membrane excitability [21]. Thus, these channels may be involved in the development of hyperalgesia and allodynia. On the contrary, spinal nerve ligation suppressed K_ATP _channels only in large-diameter neurons, but not in small-diameter neurons, from hyperalgesic rats [23].

**Figure 2:**
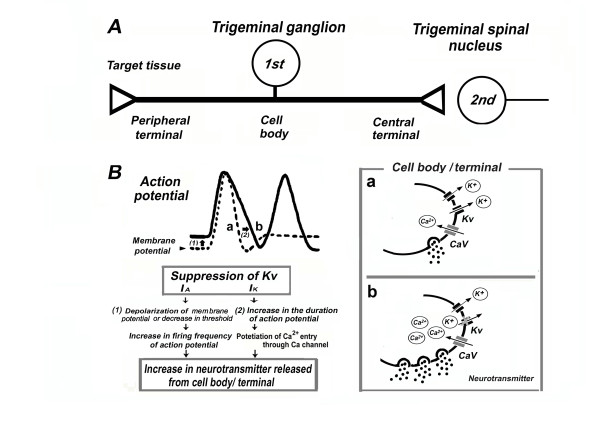
**Relationship between depression of Kv and excitability of TRG neurons under trigeminal neuropathic/inflammatory conditions**. ***A: ***The operational component of primary sensory neurons (1^st^) includes a peripheral terminal that innervates target tissues and transduces sensory stimuli. An axon conducts action potentials from the periphery to the central nervous system via the cell body in the trigeminal ganglion and central terminal, from where information is transferred to second order neurons (2^nd^) at the central synapse (e.g., trigeminal spinal nucleus). ***B: ***The relationship between the depression of Kv channels and the excitability of TRG neurons. The increases in action potential firing and duration prolongs the opening of voltage-gated Ca^2+ ^channels (Cav), potentiating Ca^2+ ^influx, and causing an increase in neurotransmitter released from the cell bodies and nerve terminals. These changes can alter the properties of trigeminal pain pathways.

As described in the preceding chapter, experiments in our trigeminal neuropathic/inflammatory pain model indicated that the common changes in the Kv channels, such as I_A_, I_K _in rats with neuropathic/inflammatory pain, were significantly suppressed compared to those in naïve rats. Since previous reports suggested that I_A _and I_K _are important for regulating the firing frequency and duration of action potentials in the TRG neuron, respectively [12,13], these Kv changes caused an incremental spike discharge and prolongation of duration of action potentials in the neuropathic/inflammatory pain rats. The relationship between depression of Kv and excitability of TRG neurons under trigeminal neuropathic/inflammatory pain conditions are summarized in Figure 2. It is well established that the duration of action potentials in primary afferents influences the amount of neurotransmitter released from the soma and the peripheral and central terminals [10,11]. Increased action potential duration and firing frequencies prolong the opening of voltage-gated Ca^2+ ^channels, probably potentiating Ca^2+ ^influx and causing an increase in the neurotransmitter released from the cell bodies and/or peripheral and central terminals (Figure 2B). Thus, enhancing the amount of neurotransmitter released from central terminals probably contributes to the hyperexcitability of second-order nociceptive and wide dynamic-range neurons in the trigeminal spinal nucleus neurons (central sensitization) [24,25,49-54]. In addition, increasing the neurotransmitters/neuromodulators (e.g., SP) released from the cell body of TRG neurons through a paracrine or autocrine mechanism may activate the neighboring TRG neurons [52,53]. Since previous studies suggested that peripheral inflammation can depolarize trigeminal satellite-glial cells via activation of neurokinin 1 receptor (SP release from small diameter TRG neurons) [55,56], such changes may further promote the interaction between satellite-glial cells and TRG neurons associated with pathological pain within the trigeminal ganglia [55-57].

The precise mechanism by which Kv depression of TRG neurons affects the neuropathic/inflammatory condition remains to be determined. Recent studies demonstrated that small- to medium-diameter TRG neurons express glial cell line-derived neurotrophic factor (GDNF) and its receptor components, such as GFRα1 [58,59]. To this end, we recently found that acute application of GDNF enhances the neuronal excitability of adult rat small-diameter TRG neurons, which innervate the facial skin in the absence of neuropathic and inflammatory conditions [60]. This potentiation of small-diameter TRG neuronal excitability is mediated by inhibition of Kv channels, and transection of sciatic nerve in rat upregulated the expression of GDNF and GFRα-1 mRNA in the proximal nerve trunk [61,62]. Thus, GDNF-induced potentiation of sensory neuronal excitability may account for enhanced pain sensitivity, resulting from increased levels of GDNF after tissue injury, and further studies are needed to elucidate possible mechanisms underlying this effect.

Finally, opening of K^+ ^channels leads to hyperpolarization of the cell membrane, and a resultant decrease in cell excitability. We thus propose the potential development of I_K _and/or I_A _channel openers as therapeutic agents for preventing trigeminal neuropathic pain, such as mechanical allodynia. Kv channel subunits, Kv2.1 and Kv2.2, contribute to the I_K _channel and play a distinct role in regulating neuronal excitability [63,64]. However, Kv1.4 and 4.1-4.3 channels may also be involved in forming A-type Kv channels in sensory neurons [65-67]. Among these Kv channel α subunits, Kv1.4, 2.2, and 4.2 mRNAs are downregulated under neuropathic conditions involving the transection and CCI of sciatic nerves [15,16]. Rasband et al. [65] demonstrated that Kv1.4 is the sole Kv α subunit expressed in smaller diameter DRG neurons, and not Kv4-family channels. Since the pharmacology and gating properties of DRG A-type Kv channels are similar to those of heterogeneously expressed Kv4 channels [66,67], we could not completely rule out a potential role for Kv4.1, 4.2, and 4.3 in neuropathic pain. Previously, we reported that TMJ inflammation decreased the expression of Kv1.4 subunits in small-/medium-diameter (Aδ-/C-) TRG neurons and that this may contribute to trigeminal inflammatory allodynia associated with TMJ disorders [68]. Nerve injury also induces accelerated reduction in Kv 1.4 expression in the small-diameter nociceptive DRG neurons [65]. Taken together, these findings suggest Kv 1.4 channel as candidate molecular targets, and further studies are needed to explore this possibility.

## Conclusion

Several different animal models of trigeminal nerve injury/inflammation showed depression of both I_A _and I_K _in TRG neurons, compared to naïve rats. These common changes contribute to the incremental spike discharge and action potential prolongation in these neurons, and may alter the properties of trigeminal pain pathways. Our results therefore would suggest that Kv channel (I_A _and I_K_) openers have potential as therapeutic agents against trigeminal neuropathic/inflammatory pain, such as mechanical allodynia.

## List of abbreviations used

4-AP: 4-aminopyridine; CCI: chronic constriction nerve; CFA: Complete Freund's adjuvant; DDP: duration of depolarization; DRG: dorsal root ganglion; DRP: duration of repolarization; α-DTX: α-dendorotoxin; FG: fluorogold; GDNF: Glial cell-deribed neruotrophic factor; I_A_: fast inactivating transient current (A-current); I _K_: dominant sustained current (K-current); IAN: Inferior alveolar nerve; ION: infraorbital nerve; K_ATP_: ATP-sensitive potassium; K_Ca_: Calcium activated potassium; Kv: voltage-gated K^+ ^channels; SP: Substance P; TEA: Tetraethylammoniun; TMJ: temporomandibular joint; TRG: trigeminal ganglion.

## Competing interests

The authors declare that they have no competing interests.

## Authors' contributions

MT conceived and wrote the manuscript. YT, JK, and KN carried out the experiments in the main part of references [24-26]. KI designed the main part of experiments [24-26]. SM conceptualized the manuscript and helped to finalize the manuscript. All authors read and approved the final manuscript.
